# WHO approves? Relative trust, the WHO, and China’s COVID-19 vaccines

**DOI:** 10.1007/s11558-022-09481-1

**Published:** 2022-11-21

**Authors:** Greg Chih-Hsin Sheen, Hans H. Tung, Chien-Huei Wu, Wen-Chin Wu

**Affiliations:** 1 Department of Political Science, National Cheng Kung University, Tainan, Taiwan; 2 Department of Political Science and and Center for Research in Econometric Theory and Applications, National Taiwan University, Taipei, Taiwan; 3 Institute of European and American Studies, Academia Sinica, Taipei, Taiwan; 4 Institute of Political Science, Academia Sinica, Taipei, Taiwan

**Keywords:** International organization, World Health Organization, Trust, Credibility, Endorsement, COVID-19 vaccines, F50, F53, O19, I18, H57, O53

## Abstract

This paper aims to answer a general question: whether an international organization (IO) is able to shape public opinion in the context of the COVID-19 pandemic. Since the pandemic took hold in early 2020, countries across the globe have switched gear from prevention to vaccination. Most had to not only secure a sufficient supply of vaccines, but also to curb vaccine hesitancy among their populations. Can endorsement by an international organization like the World Health Organization (WHO) enhance a vaccine’s acceptability? Based on a survey experiment conducted in Taiwan, our study leverages the special relationship between China and Taiwan to show that WHO endorsement can induce acceptance of Chinese vaccines among Taiwanese people. However, the effect is found to be contextual in the sense that it only works when people’s trust in the WHO is higher than their trust in the vaccine’s country of origin. Our study not only contributes to the literature of IO legitimacy by empirically showing IOs’ causal effects on public opinion, but also sheds light on how a vaccine’s credibility can be enhanced to promote vaccination uptake.

**Supplementary Information:**

The online version contains supplementary material available at 10.1007/s11558-022-09481-1.

## Introduction

Are international organizations (IOs) able to shape public opinion, either domestically or internationally, when they endorse a product or a policy of a country that lacks sufficient credibility? Over the last decade, there has been a surge in scholarly interest in IO legitimacy and legitimation (Tallberg & Zürn, 2019). On the positive side, the fact that most IOs have centralized organizations in their own professional fields and a diverse membership from different countries liberates them from parochial national interests and enables them to enjoy a good reputation internationally (Abbott & Snidal, 1998).^1^ For example, the endorsement by the United Nations (UN) has been shown to induce popular support for family policies, anti-deforestation initiatives, and the resettlement of Syrian refugees in the US (Greenhill, 2020; Linos, 2011), and also for improvements in human rights practices in Pakistan (Anjum et al., 2021).

On the negative side, however, IOs have also been found to be especially vulnerable to the undue influence by great powers. For example, the elected members of the UN Security Council have been found to receive more World Bank projects (Dreher et al., 2009) and have lower levels of conditionality attached to their IMF arrangements (Dreher et al., 2015). Moreover, these countries have also been found to give their political support to other more powerful countries in exchange for financial favors (Vreeland & Dreher, 2014). This kind of power manipulation is clearly at odds with IOs’ purported professionalism and its implications for IO legitimacy are best captured by the *guilt-by-association* argument (Guarrieri, 2018; Johnson, 2011). It suggests that one’s unfavorable view of a particular country will be readily translated into his/her view of the IOs of which this country is a member and within which it possesses greater institutionalized influence. Such a negative view undermines IOs’ alleged professional autonomy that underlies the beliefs in their credibility and legitimacy.

The two strands of the IO literature reviewed above imply that IOs are actually “Janus-faced” in having both a negative and a positive image. Consequently, their endorsement effect will have to be modulated by the relative salience of one of the two sides. To account for this duality in a single framework, we draw upon the concept of international trust (Brewer et al., 2004; Torgler, 2008) and propose in this study a trust-based perspective on IO endorsement. We tested this perspective by leveraging an experimental study on the effects of the World Health Organization (WHO) endorsement on Taiwanese people’s acceptance of COVID-19 vaccines. We found that the WHO endorsement had a positive effect on the acceptability of a vaccine when the WHO enjoyed a higher level of trust than the vaccine’s country of origin. Conversely, its effect turned negative when the country of origin was more trusted than the IO.

This study enriches the literature on IO legitimacy in the following ways. First, we engage with the growing body of literature on how IOs shape public opinion through their policy autonomy. Specifically, our experimental findings reveal that, at an individual level, the discrepancy in trust between IOs and the target of endorsement is an important condition that should be considered. Second, as China’s image had been substantially tarnished by various accusations and conspiracy theories concerning the origin of the COVID-19 virus and the Chinese government’s “wolf warrior” style of diplomacy,^2^ our experiment in Taiwan leveraged both the tension between the two sides of the Taiwan Strait and the current pandemic to empirically parse the duality of IOs’ endorsement effects. Third, our study also contributes to the literature of dictatorships by showing how they can borrow credibility from other independent sources during a crisis. Since one can hardly expect a smooth regime transition when an authoritarian country is hit by a public health crisis and its government is still the only thing its citizens can count on, from a humanitarian perspective, our findings therefore provide a temporary solution, in addition to coercive means, for both dictators and their people to weather a storm like the COVID-19 pandemic.

The rest of this article is organized as follows. We present our trust-based theory of IO endorsement in the next section, which is then followed by a section on the process by which the Chinese vaccines obtained the WHO certification and why this is relevant to the testing of our theoretical arguments. We detail our research design in Sect. 3 and present our findings in Sect. 4. In the final section, we suggest several new avenues for future research and draw conclusions.

## Theoretical argument: Relative trust and IO endorsement

The bulk of the IO literature has established the multifaceted nature of an IO’s social image, which can either be positive owing to its professionalism, or negative because of its association with a country that one distrusts. To accommodate these two aspects of an IO’s reputation within a theory of IO endorsement, we argue that the relative trust in the IO and the entity that has created the endorsed product or policy serves as a switch determining which effect becomes dominant. As Dellmuth and Tallberg (2020, 2021) demonstrate, IOs’ legitimation effect is predicated on how much people trust them, and this suggests causal heterogeneities among people with positive or negative relative trust in the IO in question.

To begin with, it is well-established in the psychology literature that people place greater weight on salient (or conspicuous) attributes (Bhatia, 2013, 2017; Bordalo et al., 2012, 2015; Kıbrıs et al., 2021). As Kahneman (2013, p.324) nicely puts it, ‘‘Our mind has a useful capability to focus on whatever is odd, different or unusual.” Our trust-based theory posits that relative trust modulates an IO’s endorsement effect by providing individuals with a cognitive shortcut that directs their attention to an IO’s salient attributes. This is particularly the case with vaccines, where the technicalities of immunology and inoculation mean that the public has only limited knowledge concerning their effectiveness, and therefore the trust in the source of vaccine-related information is highly critical (Siegrist & Cvetkovich, 2000).

From an informational perspective (Chapman, 2009; Fang, 2008), IOs’ autonomy and professionalism become more salient when they enjoy a higher level of trust among people, and their endorsement sends a costly signal to domestic audiences about the quality of either a product or a policy. If manufacturers or policymakers were not confident enough about the quality of the products or policies they are promoting, they would not have subjected them to IO scrutiny. Alternatively, if an IO is not trusted and its policy-making process is perceived to be subject to manipulation by an influential member, then the “signal” sent out through its procedures is no longer as costly and therefore less trustworthy.

In the context of the subject of this paper—that is, the WHO endorsement of a vaccine—, our argument implies that the WHO attribute that dominates its endorsement effect depends on the popular trust in the country that developed and manufactured the vaccine of interest compared to that in the WHO. When the WHO approves a vaccine developed by a country less trusted than the WHO itself, what stands out as an *unusual* feature is the WHO’s professional expertise. Alternatively, when the country of origin of a vaccine has a higher degree of credibility than the WHO, only the negative attributes of the WHO will be salient now.

We therefore present our first hypothesis as follows:

- Hypothesis 1: The WHO is expected to have a positive endorsement effect on a vaccine developed and manufactured by a country less trusted than the WHO by respondents.

In other words, Hypothesis 1 posits that individuals would prefer a COVID-19 vaccine endorsed by the WHO to one without such an endorsement when they trust the WHO more than they trust the country that developed and produced the vaccine. For example, the lack of transparency in China’s political system and its long-standing manipulation of information through its propaganda machine has tended to undermine people’s trust in the Chinese government’s endorsement of its own vaccines. Consequently, the WHO’s verification mechanism provides a natural solution to China’s credibility deficit as the WHO is arguably the most professional organization involved in handling global public health crises. Previously, it has been shown that, for its domestic audience, the Chinese government can only silence rumors and restore public trust by means of rebuttals endorsed by independent public figures (Huang, 2017). Our study further extends the argument to an international context where the endorser is an independent international organization, and the audience is foreign.

By contrast, when the trust in the WHO is lower than that in China, the opposite result is predicted. The relevance of this general argument to our study is made especially clear by Johnson (2020) and Schlipphak et al. (2022) who show both theoretically and empirically that, during an international crisis such as the COVID-19 pandemic, the IOs mandated to deal with the crisis become easy and salient targets for complaints and criticisms from citizens and elites alike in all affected countries. From this perspective, an endorsement by an IO with a lower level of trust might actually be more of a curse than a blessing. In particular, if the WHO is not trusted, its negative association with the Chinese authoritarianism—rather than its professional standing—will become much more salient since it would appear odd for a country whose public health standards have a higher level of credibility than the WHO to seek the WHO’s endorsement. If the vaccine’s efficacy and safety were not in doubt, why bother going through an additional procedure? Such an oddity, therefore, feeds suspicion that China may have undue influence in the WHO and be able to manipulate its vaccine approval procedure. Due to this “guilt by association,” we hypothesize that WHO endorsement conversely makes people more suspicious of the quality of a vaccine and lowers its acceptability. We formulate the second hypothesis of this study as follows:

- Hypothesis 2: The WHO is expected to have a negative endorsement effect on a vaccine developed and manufactured by a country more trusted than the WHO by respondents.

## China’s COVID-19 vaccines and WHO endorsement

### China’s development of COVID-19 vaccines and their global reception

As countries all over the world were battling COVID-19, a virus that had spread from China in late-January 2020, the gradual roll-out of vaccines in December that year finally allowed people to see light at the end of the tunnel. However, far from being purely a public health issue, vaccines soon became embroiled in political controversy.

China’s development of indigenous vaccines dates from well before the outbreak of COVID-19, and one of the earliest milestones in this process was recognition of its regulatory standards by the WHO, which enabled China to enter the global vaccine market (Jia & Carey, 2011). As COVID-19 originated in China, it is hardly surprising that China was one of the frontrunners in the race for a vaccine. According to information released by China’s National Medical Products Administration, as of June 7, 2021, China had granted conditional market approval to four domestic vaccines and granted emergency use authorization to one vaccine, while 21 other vaccines were going through clinical trials.

Meanwhile, unable to obtain a sufficient supply of Western vaccines, many countries had to rely on Chinese vaccines; some even accepted them at a very early stage, before the relevant data were publicly available. At the same time, it was widely reported that there was public skepticism about Chinese vaccines in many countries, developed and developing alike.^3^ Using data from a national survey they conducted in Brazil, Gramacho and Turgeon (2021) found that vaccines developed in China (and Russia) were less favored than others.

Even though all countries are desperate to secure enough doses for all their citizens so that their social and economic lives can return to normal, the decision over which vaccines to purchase is still largely determined by politics. The issue is especially salient now as China’s image has been substantially tarnished by various accusations and conspiracy theories concerning the origin of the virus and the Chinese government’s “wolf warrior” style of diplomacy. According to a study conducted by the Pew Research Center in October 2020,^4^ unfavorable views of China had reached historic highs in many countries. These opinions cast a shadow over the global acceptance of China’s vaccines. For example, President Jair Bolsonaro of Brazil remarked, ‘‘We will not buy [the vaccine] from China. It is my decision. I do not believe that it is safe because of its origin” (Gramacho & Turgeon, 2021, p. 2609).

One potential solution to the problem of political factors standing in the way of ending the pandemic is to have an internationally recognized agency act as an impartial arbiter of the efficacy and safety of vaccines. Indeed, the World Health Organization (WHO) has created a mechanism for this purpose, and various vaccines, including the Chinese ones, have been approved and endorsed by the WHO. China’s Sinopharm’s BBIBP-CorV was the first non-Western vaccine to be approved by the WHO for emergency use, and it was followed by Sinovac’s CoronaVac.

### The WHO and emergency use listing procedures

The WHO, as a specialist agency of the UN on international health matters, has a long history of involvement in vaccination campaigns, including those against smallpox and tuberculosis (Cueto et al., 2019). In the wake of the Ebola outbreak of 2014–16, the WHO decided to establish an Emergency Use Assessment and Listing (EUAL) procedure to temporarily grant approval for unlicensed vaccines, medicines, and in vitro diagnostics (IVDs) in the event of public health emergencies—that is, the outbreak of a highly transmissible disease or one with a high mortality rate and no available treatment or prevention (WHO, 2020, p. 8). However, the procedure suffered from poor quality of submissions and validation data, a lack of international standards to guide the assessment, insufficient reference preparation and panels for validating assays, the lack of an ethical code governing the source of materials, and problems with the biosafety of IVDs, all of which demanded the development of a better procedure (WHO, 2020, pp. 7–8).

To improve on the existing EUAL procedure, the WHO decided to reframe it as the Emergency Use Listing (EUL) procedure. EUL is primarily designed for a public health emergency of international concern (PHEIC) and, what is particularly important, the use of an unlicensed product under the EUL framework is based on a predetermined rationale and predetermined criteria (WHO, 2020, pp. 8–9). EUL offers a 12-month listing to assist interested UN procurement agencies and member states in deciding whether to use particular products according to an essential set of available quality, safety, and efficacy data, regardless of the limited amount of data available and the products not being ready for prequalification application (WHO, 2020, p. 16).

Upon receiving an application from a manufacturer in the event of a public health emergency, the WHO will form two agencies, the Product Evaluation Group (PEG) and the Advisory Group on Emergency Listing (TAG-EUL), from the roster of experts provided by the Regulation and Prequalification Department (RPQ). These agencies are responsible for setting the standards for the listing and acceptability of certain products. The PEG will consider and review international and national standards and the scientific literature and assess the safety or efficacy of the products in question. The TAG-EUL will then decide on the acceptability of those products according to the standards. Listing decisions, related assessments, and communications (even negative results) will be made public on the WHO website once the RPQ makes its decision (WHO, 2020, pp. 10–15). After a product is listed, the WHO will monitor its performance. However, the WHO makes it clear that “Member States have the sole prerogative to use the EUL as the basis to authorize the use of an unlicensed vaccine/medicine/IVD at the national level” (2020 p. 9), even though national authorities give great weight to the EUL.

### The COVAX facility

When the WHO declared COVID-19 to be a PHEIC on January 30, 2020, the international community was faced with the challenge of developing and distributing effective vaccines for all those in need of them. In April 2020, the WHO, the European Commission, and the French government launched the Access to COVID-19 Tools (ACT) Accelerator that was aimed at bringing together national governments, intergovernmental organizations, nongovernmental organizations, civil society, and the private sector to work out how to provide equitable access to COVID-19 diagnostics, treatments, and vaccines. The COVID-19 Vaccine Global Access Facility (COVAX) constitutes the vaccine pillar of the ACT Accelerator, and it aims to ensure equitable access to COVID-19 vaccines, prioritizing high-risk and vulnerable people. Its target was to make available two billion doses by the end of 2021 (COVAX 2020). COVAX is co-led by the Vaccine Alliance (GAVI), the WHO, and the Coalition for Epidemic Preparedness Innovations (CEPI) and administered by GAVI. Its main objective is to “maximize … chances of successfully developing Covid-19 vaccines and manufacture them in the quantities needed to end this crisis, and in doing so ensure that ability to pay does not become a barrier to accessing them” (Berkeley, 2020). To this end, COVAX is securing supplies of vaccines through advanced market commitments by leveraging the amount committed by high-income countries while at the same time enabling the participation of low-income states. EUL by the WHO is a prerequisite for inclusion in COVAX.

In this article, we argue that WHO approval and inclusion in COVAX would make a COVID-19 vaccine more acceptable to the public. Specifically, since ordinary citizens do not have sufficient expertise to understand the efficacy and potential side-effects of vaccines, they would tend to rely on the WHO’s expertise. However, the endorsement effect is conditional on an individual’s trust in the WHO relative to their trust in the country of origin of the vaccine.

### Skepticism about China’s COVID-19 vaccines in Taiwan

While China has made extensive efforts to develop and promote its vaccines, concerns nonetheless persist as to their safety and efficacy. Doubts about the safety and efficacy of Chinese drugs, vaccines included, have a long history as the result of numerous scandals, such as the 2018 Changsheng Bio-technology vaccine fraud case (Cheung, 2013; McLaughlin, 2016; Zhou et al., 2019). For this reason, some foreign consumers have concerns about accepting Chinese COVID-19 vaccines, especially when China persistently declined to publish results of its phase III clinical trials. It was not until May 26, 2021, that Sinopharm published its clinical trial results, but information on the effects of the vaccine on clinically vulnerable groups remained missing (Al Kaabi et al., 2021). For these reasons, regardless of the price advantage and ready availability, skepticism about China’s vaccines persists. Most developed countries, such as those of the European Union (EU), the US, and Japan, have not granted market approval to Chinese vaccines. Therefore, China is keen to seek recognition for its indigenous vaccines from the international organization responsible for global health, namely, the WHO. Among the approved or authorized vaccines, two—Sinopharm’s BBIBP-CorV and Sinovac’s CoronaVac—have been granted EUL by the WHO.

Chinese COVID-19 vaccines have attracted even more skepticism in Taiwan than in other countries. China sees Taiwan as a breakaway province, and relations between the two sides have deteriorated since the pro-independence Democratic Progressive Party (DPP) candidate, Tsai Ing-wen, won the 2016 presidential election and rejected the “1992 Consensus.”^5^ Since then, China has coerced seven countries to cut off diplomatic ties with Taiwan, reducing the number of Taiwan’s formal diplomatic allies from 22 to 15. Since 2017, China has blocked Taiwan’s participation in the World Health Assembly (WHA) annual meeting. As China continues to restrict Taiwan’s international space, “made-in-China” products have fallen out of favor in Taiwan, and Chinese COVID-19 vaccines are no exception (BBC, 2020). For example, Taiwan’s attempted purchase of Pfizer-BioNTech COVID-19 vaccines was controversial because the vaccines are distributed by a Chinese company, Shanghai Fosun Pharmaceutical.

Thanks to Taiwan being a relatively small island, it was not too difficult to control inbound visitors. As of March 2021, there had been just over one thousand cases of COVID-19 in Taiwan, with only 10 deaths. Ng et al. (2021) suggest that Taiwan’s early success in combating COVID-19 was due to its effective use of both case-based interventions, such as contact tracing and quarantine, and population-based interventions, such as social distancing and the wearing of face masks. Despite the experts’ prediction that Taiwan would have the world’s second highest number of COVID-19 cases after China (Wang et al., 2020), life on the island remained normal; no lockdown measures were introduced and the vast majority of businesses remained open.

Taiwan remained almost COVID-19-free until mid-May 2021. After the quarantine period for non-vaccinated airline pilots was shortened from two weeks to three days, there was an outbreak of COVID-19 in the Novotel at Taoyuan International Airport where the pilots were required to quarantine. From there, the virus quickly spread island wide (Aspinwall, 2021), and the numbers of confirmed COVID-19 cases and related deaths surged. As the public became more worried about contracting the virus, Taiwan struggled to obtain vaccines and the domestic rollout was slow.

Concerned about the central government’s failure to obtain supplies of vaccines from overseas and the slow development of domestic ones, many people, including entrepreneurs and local government heads, began sourcing vaccines elsewhere. According to a report in China’s *Global Times,* more and more Taiwan residents, including political figures, lost faith in the incumbent party, “so they came to the Chinese mainland to seek solutions such as getting vaccinated or purchasing Chinese mainland-made vaccines” (Fan et al., 2021). While this claim is hardly mainstream, it offers a perspective on the behavior of some people on the island. Moreover, two Chinese vaccines, those produced by Sinopharm and Sinovax, obtained WHO approval in June 2021, making the option of accepting Chinese vaccines a real subject of debate. While it is unlikely that the Tsai administration will introduce Chinese vaccines into Taiwan (Davidson, 2021), it is theoretically important and politically relevant to examine how the public in Taiwan views Chinese vaccines and how WHO approval affects their views.

Due to skepticism about the WHO’s management of COVID-19 and the history of antipathy between Taiwan and the WHO, we argue that Taiwan serves as a critical case when investigating the effect of WHO endorsement on COVID-19 vaccines. We are naturally interested in learning how WHO approval of Chinese vaccines would affect the Taiwanese public’s acceptance of them. As Larson (2018) suggests, because vaccines are regulated, and sometimes mandated, by governments, vaccination tends to be resisted by those who feel that their personal freedom is being encroached upon and by those who do not trust the government for reasons that may be unrelated to vaccines themselves. Suggesting that the Taiwan context may be the least likely to produce a favorable finding, we explore how trust in an international organization may help improve the public’s attitude toward products from an unpopular country.

## Research design

To test our hypotheses, we conducted a survey experiment involving a diverse sample of 950 Taiwanese adults from May 23 to June 6, 2021. The sample was recruited by Rakuten Insight, an international public opinion company that conducts online surveys in Asian countries. The sample reflected the composition of the adult population of Taiwan in terms of gender, age, and geographic distribution. The survey began just four days after Taiwan declared a nationwide COVID-19 Level 3 alert. Taiwan had registered a total of 1,132 confirmed COVID-19 cases between early 2020 and May 1, 2021, but by May 22, that number had soared to 3,862 (a roughly 341% increase). There were 7,584 additional confirmed cases and 170 deaths during our survey period. With the sudden surge in cases and deaths, the question of when and how the government would procure enough vaccines for its citizens had become extremely salient. We utilized this window of opportunity to explore public support for government procurement of foreign vaccines before those vaccines arrived in Taiwan. In other words, our results were not contaminated by the actual procurement of the vaccines. More importantly, because of Taiwan’s relations with China and the WHO, we believe that Taiwan is an ideal case to test our hypotheses on the endorsement effect of the WHO on COVID-19 vaccines.

Our survey was divided into three parts. First, the subjects were asked some demographic questions, including their gender, year of birth, level of education, and area of residence. Second, they were asked questions designed to investigate their attitudes toward COVID-19 and their overall level of trust in the WHO and five countries, namely, Taiwan itself, the US, Germany, Russia, and China. We also asked them to evaluate the overall performance of the WHO and these countries in containing the COVID-19 pandemic. The operationalization and summary statistics of variables used in the following empirical analysis are presented in Tables A.1 and A2, respectively, in the supplementary material.^6^

**Table 1:** Estimates of Support for Government Procurement of COVID-19 Vaccines from Abroad

	Model 1	Model 2	Model 3	Model 4
	Chinese Vaccines		Foreign Vaccines	
Age	0.019*	0.020*	0.0004	0.0003
	[0.010]	[0.010]	[0.014]	[0.014]
Female	-0.317	-0.318	0.398	0.407
	[0.216]	[0.216]	[0.376]	[0.370]
College	0.08	0.081	0.744*	0.775*
	[0.222]	[0.223]	[0.367]	[0.374]
Willingness	-0.012	-0.011	0.228***	0.235***
	[0.041]	[0.041]	[0.067]	[0.069]
Support Incumbent	-1.331***	-1.307***	-0.598	-0.629
	[0.373]	[0.371]	[0.490]	[0.494]
Nationalism	-0.564***	-0.569***	0.005	0.01
	[0.127]	[0.128]	[0.272]	[0.270]
WHO Approval	0.347 +	0.178	-1.455***	-1.052*
	[0.209]	[0.243]	[0.410]	[0.501]
Trust Differential	-0.182	-0.350 +		
(WHO-China)	[0.138]	[0.202]		
WHO Approval X		0.36		
Trust Differential (WHO-China)		[0.284]		
Trust Differential			0.219	-0.131
(WHO-the US)			[0.181]	[0.326]
WHO Approval X				0.451
Trust Differential (WHO-US)				[0.400]
Constant	0.661	0.677	1.567	1.158
	[0.621]	[0.625]	[1.211]	[1.255]
Log pseudolikelihood	-272	-271	-114	-113
No. of Observations	441	441	438	438

Models 1 and 2 pool the samples of the Chinese vignette (China vs. China + WHO), while Models 3 and 4 pool the samples of the foreign vignette (Foreign vs. Foreign + WHO). Robust standard error in brackets. + 0.1, * *p* < 0.05, ** *p* < 0.01, *** *p* < 0.001. All tests are two-tailed

**Table 2:** Correlates of Trust and Trust Differentials

	Model 1	Model 2	Model 3	Model 4	Model 5
	China	US	WHO	WHO-China	WHO-US
Age	0.002	0.002	-0.001	-0.004 +	-0.003
	[0.002]	[0.002]	[0.002]	[0.002]	[0.003]
Female	-0.003	0.039	0.072 +	0.05	0.021
	[0.040]	[0.041]	[0.039]	[0.048]	[0.056]
College	-0.057	0.057	-0.008	0.021	-0.074
	[0.041]	[0.043]	[0.042]	[0.052]	[0.058]
Nationalism	-0.021	0.064*	-0.026	0.02	-0.073*
	[0.024]	[0.027]	[0.024]	[0.029]	[0.036]
Transparency-China	0.375***				
	[0.041]				
Performance-China	0.282***				
	[0.029]				
Transparency-US		0.392***			
		[0.035]			
Performance-US		0.272***			
		[0.029]			
Transparency-WHO			0.387***		
			[0.037]		
Performance-WHO			0.388***		
			[0.042]		
Transparency Differential				0.305***	
(WHO -China)				[0.032]	
Performance Differential				0.232***	
(WHO -China)				[0.030]	
Transparency Differential					0.472***
(WHO- US)					[0.037]
Performance Differential					0.332***
(WHO-US)					[0.040]
Constant	0.474***	0.755***	0.535***	0.311*	0.042
	[0.124]	[0.151]	[0.123]	[0.128]	[0.158]
R-squared	0.473	0.308	0.535	0.277	0.439
No. of Observations	858	849	834	806	803

Robust standard error in brackets. + 0.1, * *p* < 0.05, ** *p* < 0.01, *** p < 0.001. All tests are two-tailed

The third part of the survey consisted of the experiment. As indicated in our hypotheses, we adopted a 2 × 2 factorial design in this study. We began by asking respondents to indicate their willingness to be vaccinated against COVID-19 on a scale of 0–10, with 10 indicating the greatest willingness. We then randomly assigned each respondent to one of the following four groups and presented them with different vignettes, which varied in terms of the origins of the COVID-19 vaccines and whether the WHO had approved them. Group 1, the “foreign^7^ vaccines group,” was asked to read the following paragraph:

*Scientists have developed COVID-19 vaccines, and our government is beginning to procure COVID-19 vaccines from abroad for your fellow countrymen. Do you support procurement by our government of COVID-19 vaccines developed by foreign countries?*

The respondents were asked to indicate their support on a four-point Likert scale, with 1 indicative of “strongly do not support,” 2 of “somewhat do not support,” 3 “somewhat support,” and 4 “strongly support.”

Group 2 (the “Chinese vaccines group”) was presented with an almost identical vignette, but the words “vaccines developed by foreign countries” was replaced with “vaccines developed by China.” This group was asked to indicate their support on the same scale used for Group 1.

The first two vignettes focused on the origins of the COVID-19 vaccines. We then added another attribute of COVID-19 vaccines: WHO approval. Specifically, Group 3 (“foreign vaccines + WHO”) was asked to read the following:

*The World Health Organization (WHO) is using COVID-19 Vaccines Global Access (COVAX) to provide countries with COVID-19 vaccines that it has approved. Do you support procurement by our government of COVID-19 vaccines developed by foreign countries and approved by the WHO?*

Again, respondents were asked to express their support on a four-point Likert scale, with a higher value indicative of greater support.

Group 4 (“Chinese vaccines + WHO”) was shown the following and asked to indicate their support on the same scale:

*The World Health Organization (WHO) is using COVID-19 Vaccines Global Access (COVAX) to provide countries with COVID-19 vaccines that it has approved. In May 2021, the WHO approved the Sinopharm vaccine developed in China and included it in COVAX. Do you support procurement by our government of China’s COVID-19 vaccines approved by the WHO?*

We collected 950 successful interviews in our study, with about 240 respondents in each group. The balance table of key variables is presented in Table A.3 in the supplementary material. This indicates that our random assignment is successful in terms of respondents’ demographic traits and political attitudes. We also include a transcription of the questionnaire used in this study in Sect. 4 of the supplementary material.

### Main results

We first investigated whether respondents’ support for government procurement differs according to the origin of the COVID-19 vaccines. We expected Taiwanese to express more opposition to the procurement of vaccines developed by China than to the procurement of foreign vaccines in general. Similarly, we predicted that respondents would be less supportive of the procurement of Chinese vaccines approved by the WHO than they would of WHO-approved foreign vaccines. In addition to the national origin of the vaccines, we were also interested in whether there would be more support for a COVID-19 vaccine that was approved by the WHO and included in COVAX. Following our theory, we expected that both foreign and Chinese vaccines would get more or less support if respondents knew that they were approved by the WHO depending on the relative trust between the WHO and the vaccine’s country of origin.

**Fig. 1:**
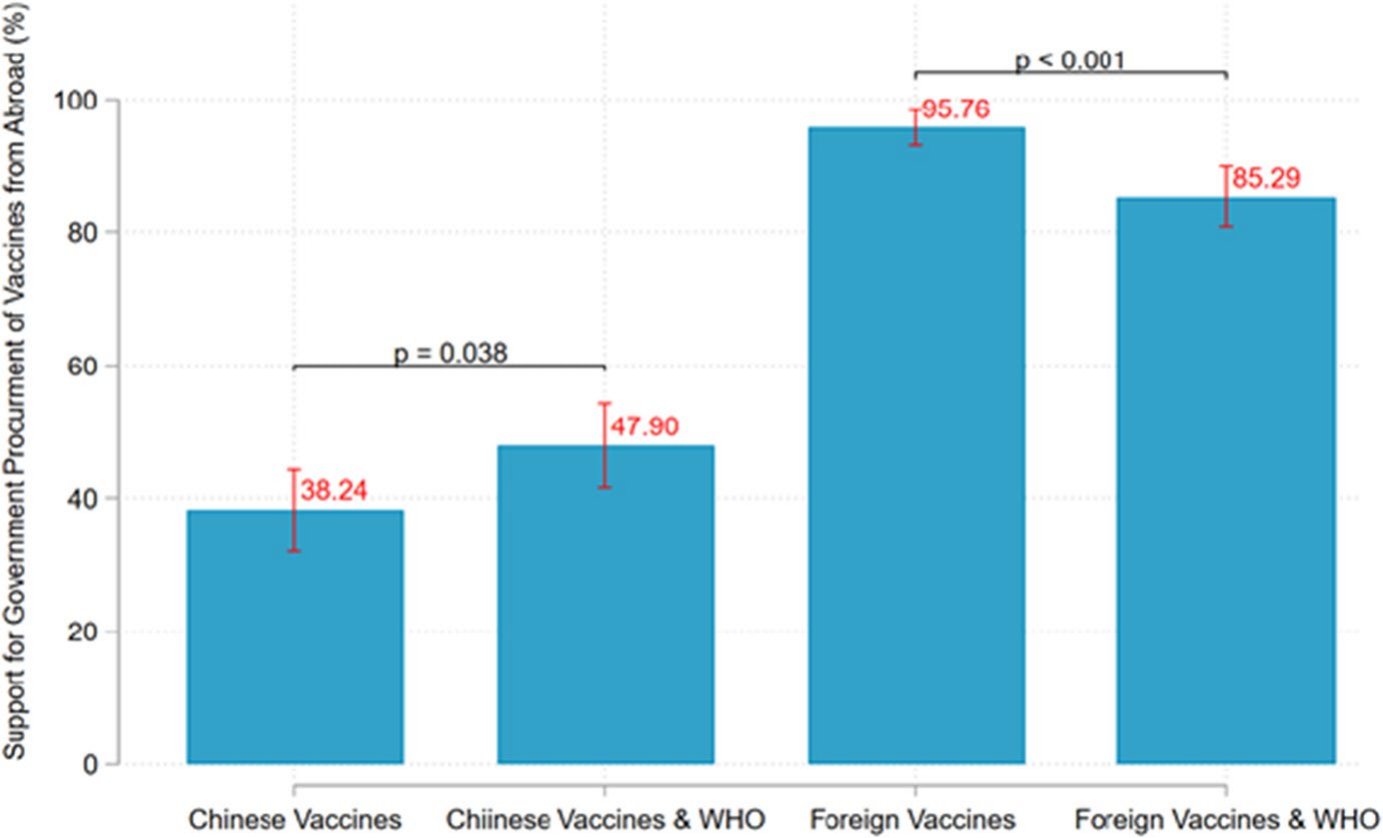
Support for Government Procurement of COVID-19 Vaccines

Figure 1 further visualizes the distribution of respondents’ support for the procurement of foreign vaccines. For ease of interpretation, we coded the answers “strongly do not support” and “somewhat do not support” as 0, and “somewhat support” and “strongly support” as 1.^8^ Accordingly, the height of each bar in Fig. 1 displays the average support rate for each kind of vaccine, with the vertical lines being 95% confidence intervals. From Fig. 1, we can see that the respondents were most supportive of the procurement of foreign vaccines (95.76%) and least supportive of Chinese vaccines (38.24%).^9^

Although Chinese vaccines are less popular than foreign vaccines, Fig. 1 further suggests that WHO approval does induce more support for Chinese vaccines. Specifically, the support rate of Chinese vaccines approved by the WHO is 47.90%. This is 9.42 percentage points higher than the support rate for Chinese vaccines within the “Chinese vaccines group” (which received no information concerning WHO approval), and the difference between the groups is statistically significant (p = 0.038). The result is consistent with our expectation that WHO approval would make Chinese vaccines more acceptable to our respondents.

Moreover, Fig. 1 indicates that the support rate for WHO-approved foreign vaccines is 10.47 percentage points lower than that for foreign vaccines in general (95.76% vs. 85.29%, p < 0.001). In other words, WHO approval does not induce support for foreign vaccines but actually reduces it. While this result is somewhat counterintuitive, it is consistent with our theoretical expectation for the relationship between differential trust and vaccine support. Based on our theory discussed in the previous section, we would expect WHO endorsement to increase support for a vaccine only when respondents trust the WHO more than they trust the vaccine’s country of origin. In the next section, we further investigate this hypothesis on the relationship between trust differentials and support for WHO-endorsed vaccines.

### The effects of trust differential on support for COVID-19 vaccines

In the previous section, we found that Chinese vaccines are less preferred than foreign vaccines, regardless of whether they are approved by the WHO. Yet, Chinese vaccines become more acceptable if they have WHO approval. Meanwhile, foreign vaccines in general become less popular if respondents learn that they are approved by the WHO. Our theory suggests that it is difference in trust between China, foreign countries, and the WHO that causes the different levels of support for vaccines developed by China and by foreign countries with or without WHO approval. To empirically test our argument, we use the responses to the pre-treatment questions on trust in China, foreign countries, and the WHO. Specifically, we asked respondents before they were presented with the experimental vignettes to indicate their trust in five individual countries (Taiwan, China, the US, Germany, and Russia) and their trust in the WHO on a 1–4 Likert scale, with 1 being indicative of “very untrustworthy,” 2 “somewhat untrustworthy,” 3 “somewhat trustworthy,” and 4 “very trustworthy.” It should be noted that in the following analysis, we use respondents’ trust in the US as an indicator of their trust in foreign countries, because the US was the key provider of COVID-19 vaccines to Taiwan when we conducted the survey. Nevertheless, our results remain unchanged if we use respondents’ trust in Germany, another key provider of COVID-19 vaccines at that time (i.e., the Pfizer-BioNTech), as an alternative indicator of trust in foreign countries.^10^ We present these results in Table A.6 and Figure A.2 in the supplementary material.

**Fig. 2:**
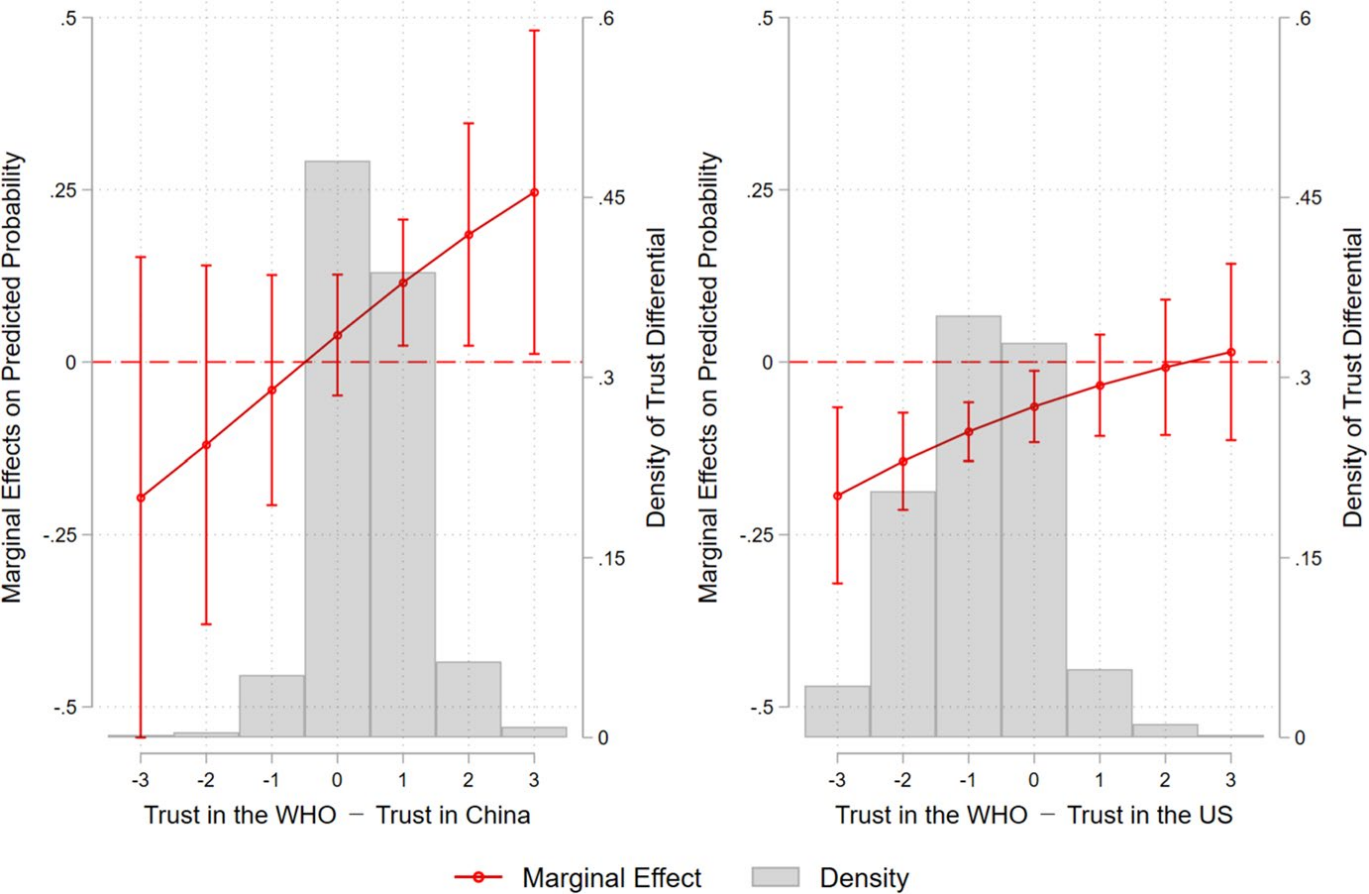
Marginal Effect of WHO Approval on Support for Government Procurement of Vaccines from Abroad across Trust Differentials. Note: The figures are based on the estimation of Models 2 and 4 in Table 1, respectively. The x-axis of panel (**a**) measures the credibility deficit/surplus of the WHO by subtracting respondents’ trust in China from their trust in the WHO. The x-axis of panel (**b**) measures the credibility deficit/surplus of the WHO by subtracting respondents’ trust in the US from their trust in the WHO. Vertical lines indicate 90% confidence intervals

Based on respondents’ answers to questions concerning their trust in China, the US, and the WHO, we create two variables that measure the differences between their trust in the WHO and China as well as the US. Both measurements of trust differentials, Trust in WHO-Trust in China and Trust in WHO-Trust in the US, range from -3 to 3, with higher numbers indicative of more trust in the WHO relative to China and the US, respectively.

To further examine the effects of trust differentials on respondents’ support for government procurement of vaccines from abroad, we estimate a series of logit models, with 0 indicating that respondents “strongly do not support” or “somewhat do not support” government procurement of vaccines developed by China, and 1 indicating that respondents “somewhat support” or “strongly support” such procurement. In addition to the measurements of trust differentials, we include other variables that may confound the relationship between trust differentials and vaccine support, such as respondents’ age, gender, education level, support for the incumbent government, and nationalistic sentiment. Again, Tables A.1 and A.2 in the supplementary material present the operationalization and summary statistics of these variables, respectively.

Using the variables of trust and other control variables, we first estimate two models by pooling our respondents in the Chinese vaccines group and the Chinese vaccines with WHO approval group. We create a dummy variable to indicate whether a respondent is in the Chinese vaccines group or in the Chinese vaccines with WHO approval group. Results of Model 1 in Table 1, consistent with Fig. 1, show that respondents in the latter group are more supportive of government procurement of vaccines from China. As we are concerned whether the trust differential between the WHO and China influences respondents’ support for vaccines, in Model 2 we interact a variable of trust differential and the dummy of WHO approval. We follow the methodological advice of Berry et al. (2012) and draw a marginal effect plot to illustrate how the marginal effect of WHO approval changes over the range of respondents' trust differentials between the WHO and China. As Fig. 2(a) demonstrates, the marginal effect of WHO approval on respondents’ support for Chinese vaccines becomes positive and statistically significant at the p < 0.1 level when their trust in the WHO is higher than their trust in China. In other words, if a respondent’s trust in the WHO is higher than her trust in China (i.e., the trust differential is positive), then WHO approval would increase her support for Chinese vaccines.

We repeated the same estimation procedure for the foreign vaccines and foreign vaccines with WHO approval groups. The results are reported in Models 3 and 4 in Table 1. We also drew a marginal effects plot to illustrate the effects of WHO approval on foreign vaccines across respondents’ differences in trust in the WHO and the US. Figure 2(b) shows that when a respondent’s trust in the WHO is the same or less than her trust in the US (i.e., trust differential is 0 or negative), WHO approval decreases her support for a foreign vaccine. We should like to highlight that that the correlation between respondents’ trust in China and their trust differential between the WHO and the US is 0.438. This moderate correlation indicates that when a respondent has low trust in China, her trust differential between the WHO and US would be negative as well. In other words, respondents’ trust in China may “spill over” to their trust in the WHO.^11^ This finding further substantiates our argument about the effect of relative trust on WHO endorsement.

It is noteworthy that Figs. 2(a) and 2(b) show consistent and complementary results. In the case of Chinese vaccines, WHO approval induces vaccine support when respondents’ trust in the WHO is higher than their trust in China, whereas in the case of foreign vaccines, respondents’ support is lower if their trust in the WHO is no greater than their trust in the US. In other words, the effect of WHO approval is mainly driven by more trust (distrust) in the WHO than in China (or the US) among respondents in the groups receiving vignettes of Chinese (or foreign) vaccines. These findings offer empirical support for our hypotheses on the trust-based theory of IO endorsement. In addition, Fig. 2(b) suggests that for foreign vaccines, WHO endorsement exerts no additional positive effect on respondents’ support, because support is already quite high. Put differently, there is a “ceiling effect” of trust in foreign countries on WHO-approved vaccines. Similarly, there is a “floor effect” of trust in China for Chinese vaccines in Fig. 2(a), as very few respondents have a higher level of trust in China than in the WHO.^12^

The results for some control variables in our empirical models are worthy of discussion, too. First, Models 1 and 2 show that respondents who are more supportive of the incumbent government or are more strongly nationalistic are less supportive of Chinese vaccines. Both results are consistent with the stylized facts that the incumbent government is more anti-China and that more strongly nationalistic Taiwanese are also more anti-China. Yet, neither support for the incumbent nor strong nationalistic sentiment is statistically significant in Models 3 and 4 for foreign vaccines, suggesting that the two variables play an important role in shaping respondents’ support for Chinese vaccines, but not for foreign vaccines. Meanwhile, Models 3 and 4 indicate that respondents with a college degree are more likely to support foreign vaccines than those without a degree. Those who are more willing to be vaccinated are also more supportive of foreign vaccines than those who are less willing to be vaccinated. Nevertheless, we find no evidence that respondents’ education level or willingness to be vaccinated affects their support for Chinese vaccines.

### Sources of trust in China, foreign countries, and the WHO

In the previous section, we showed that the endorsement effect of WHO approval on COVID-19 vaccines depends on the trust differentials between the WHO and the countries of origin of the endorsed vaccines (i.e., China and other foreign countries). One may wonder what the measurements of trust and trust differentials exactly measure in our study. As we regard a respondent’s trust in a specific country or the WHO as a summary measure, or a “short cut” for their evaluation of foreign countries, in this section we further investigate the correlates of individual trust in these countries and the WHO.

We argue that citizens’ trust in foreign countries and the WHO indicates their evaluation of the credibility of these countries and the WHO. This perceived credibility would be correlated with respondents’ perception of these countries’ transparency where information about the COVID-19 pandemic is concerned and their performance in dealing with the pandemic. More specifically, a country that is less willing to release information about the pandemic or deals with the pandemic in an unsatisfactory way would be seen as less trustworthy by citizens of foreign countries. To empirically test this claim, we have created variables based on respondents’ evaluations of transparency concerning COVID-19 and performance in dealing with the pandemic for China, the US, and the WHO, respectively. To do this, we asked respondents two questions before presenting them with the experimental vignettes: (1) What is the level of transparency of the following countries and the WHO when they release information regarding the COVID-19 pandemic? and (2) What is your opinion on the performance of different governments and the WHO in addressing the COVID-19 outbreak? Respondents were asked to offer their evaluations on a 1–4 Likert scale (i.e., not transparent at all to very transparent in the case of question 1 and very bad to very good for question 2).

**Fig. 3:**
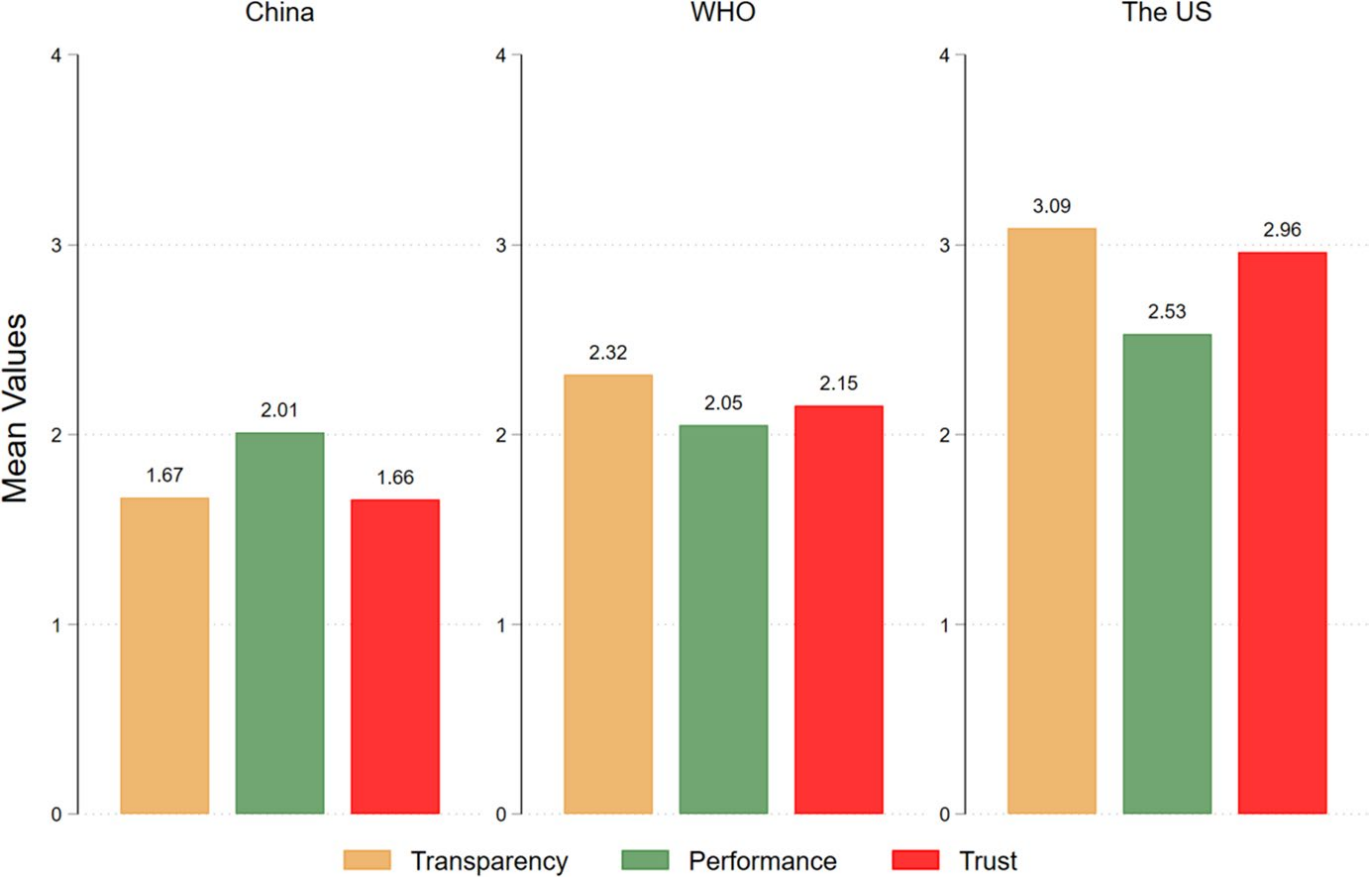
Transparency regarding COVID-19 Information, Performance in Dealing with COVID-19, and Trust in Individual Countries and the WHO

Figure 3 is a bar chart of respondents’ answers to the questions on transparency and performance, and their trust in China, the WHO, and the US. Figure 3 conveys two key messages. First, respondents’ evaluation of the transparency and performance of both China and the WHO is lower than their evaluation of the transparency and performance of the US. Second, their evaluation of transparency and performance is correlated with trust, which is higher for the US than it is for China or the WHO. In other words, there is a hierarchy of trust in foreign countries and the WHO among our respondents, and this trust is correlated with respondents’ evaluation of the COVID-related transparency and performance of foreign countries and the WHO.

To further investigate the relationship between respondents’ trust in foreign countries and their evaluation of those countries’ COVID-related transparency and performance, we estimated OLS models that regress the trust variables on the variables of transparency and performance. We also included respondents’ demographic characteristics, such as age, gender, and education level. The results, as shown in Models 1 to 3 in Table 2, suggest that a country’s (or the WHO’s) transparency and performance are both positively correlated with respondents’ trust in that country (or the WHO). In Models 4 and 5, we further used the two variables of trust differential constructed in the previous section and regressed them on the transparency and performance differentials between the WHO and China as well as the US, respectively. The results indicate that when respondents think that the WHO is more transparent or is doing a better job in dealing with the pandemic than China (or the US), they have a higher level of trust in the WHO than in China (or the US).^13^ In summary, respondents’ trust in China, the US, and the WHO is correlated with their evaluation of the transparency and performance of these international actors. This finding further supports our use of trust to explain respondents’ support for government procurement of COVID-19 vaccines from abroad.

## Conclusion

In this article, we address the issue of whether IOs can shape public opinion. Leveraging variations in trust in COVID-19 vaccines, we conducted a survey experiment in Taiwan aimed at understanding the effect of the WHO’s endorsement of a vaccine. Our results show that while, on average, the WHO endorsement has a positive causal effect on the acceptance of vaccines developed by China, a country considered to be lacking in credibility by the majority of the survey respondents, the endorsement effect was heterogeneous—i.e., it was positive among those who placed greater trust in the WHO than they did in China, but negative among those whose trust hierarchy was reversed. This confirms our trust-based theory of IO endorsement. Moreover, we also found that, for vaccines developed in the West, WHO endorsement as a treatment exerted no positive effect on their acceptability since they were already extremely popular among Taiwanese when the experiment was conducted.

Our findings contribute to the literature in several ways. First, they enrich our understanding of the Janus-faced nature of IOs and how this affects IOs’ ability to shape public opinion through endorsement, especially during a crisis like the current pandemic. Second, this study contributes to the literature on comparative authoritarianism by showing how dictatorships can borrow credibility from independent sources during a crisis. Even though Taiwanese citizens’ distrust of China made Chinese vaccines unpopular in Taiwan, our empirical findings suggest that the WHO’s verification mechanism, when the WHO was trusted, provided a (partial) solution to China’s credibility deficit. Since authoritarian regimes often lack credibility for their opaqueness, they would be advised to actively utilize international verification mechanisms to convince external parties.

Finally, our study also opens at least two new avenues for future research. First and foremost, as we only focused on the acceptability of COVID-19 vaccines during a public health crisis and the role played by the WHO endorsement, a natural next step would be to investigate whether our trust-based theory of IO endorsement also applies to other issue areas (e.g., financial crises) and other IOs (e.g., the IMF or the World Bank). Second, besides IOs, as Sheen et al. (2021) show experimentally in the same context of the COVID-19 pandemic, China could alternatively borrow credibility from independent citizen journalists who could facilitate its risk communication. Following this line of empirical investigations, future research should explore other independent sources of credibility (e.g., international ratings agencies or academic institutions) to see whether there is any variation in their effectiveness. This study also has practical implications for IOs’ legitimation strategy (Gronau & Schmidtke, 2016; Von Billerbeck, 2022) and the elite communication approach to IO legitimacy pioneered by Dellmuth and Tallberg (2021). Since the relative trust between the IO of interest and the maker of an endorsed product or policy can be a significant predictor of the former’s endorsing effect, it provides a useful guideline for elites to tailor messages in their communication with particular groups. They should keep in mind that IOs are Janus-faced and their endorsements can be a blessing to some but a curse to others.

## Supplementary Information

Below is the link to the electronic supplementary material.

Supplementary file1 (DOCX 267 KB)

Supplementary file2 (zip 40.0 KB)

## Data Availability

The data that support the findings of this study are available from the corresponding author upon request and will be uploaded to the Review of International Organizations’ website after publication.

## Footnotes

1 The credibility of IO endorsement is also enhanced by IOs’ expertise in highly technical domains. Since most national leaders are generalists whereas many IO professionals have appropriate formal (graduate-level) qualifications and experience gained in their pre-IO careers, the latter are seen as trusted sources of information, independent of national special interests. In addition to the qualifications of IO professionals, most IOs have well-received and transparent procedures for their independent quality verification processes, so their professional reports and publications often serve as important references for local actors in individual countries.

2 According to a study conducted by the Pew Research Center in October 2020 (https://www.pewresearch.org/global/2020/10/06/unfavorable-views-of-china-reach-historic-highs-in-many-countries/), unfavorable views of China had reached a historic high in many countries. These opinions would have cast a shadow over the global acceptance of China’s vaccines.

3 For related news reports see: https://www.abc.net.au/news/2021-01-22/china-coronavirus-covid-19-vaccine-equity-sinovac-sinopharm/13066856, https://www.abc.net.au/news/2021-03-26/why-is-the-west-so-sceptical-of-chinas-vaccines-/100026036, and https://www.voanews.com/a/covid-19-pandemic_chinas-vaccine-sent-developing-nations-may-find-wary-reception/6203620.html.

4 The survey results are available online at: https://www.pewresearch.org/global/2020/10/06/unfavorable-views-of-china-reach-historic-highs-in-many-countries/.

5 The “1992 consensus” is a tacit understanding between the Chinese Nationalist Party (Kuomintang, KMT) and Beijing that both sides of the Taiwan Strait acknowledge that there is only “one China,” with each side having its own interpretation of what “China” means. Su Chi, the former chairman of Taiwan’s Mainland Affairs Council, claimed in 2006 that he had coined the term in 2000.

6 Supplementary material for this article is available on the website of the Review of International Organizations.

7 We used the term “foreign” here instead of naming a specific country for two reasons: First, apart from the Chinese COVID-19 vaccines, the most discussed vaccine in Taiwan at the time of the experiment was the Pfizer-BioNTech, which was an mRNA-based vaccine jointly developed by American and German companies. As a result, the contrast in Taiwanese society then was between China and foreign countries as a group rather than between China and a specific third country (see a related report: https://www.taiwannews.com.tw/en/news/4276952?fbclid=IwAR2slag8cBdq-Lzwlu7ceU5AwzfYdygsy8MD-kj530BYx9up9AJEZN_cnvI). More importantly, Taiwanese people tend to regard most foreign, especially Western, countries as a fairly homogeneous group in this regard. Second, as our survey results show, most foreign countries enjoy similar levels of trust in Taiwan. Consequently, Taiwanese people’s responses to the generic category of “foreign countries” should not be very different from their response to an individual country such as the US or Germany.

8 Our results remain unchanged when we use the original level of support and calculate the average support for each group.

9 Table A.4 in the supplementary material shows the distribution of respondents’ support for government procurement of different COVID-19 vaccines.

10 See the following report: https://www.taiwannews.com.tw/en/news/4276952?fbclid=IwAR2slag8cBdq-Lzwlu7ceU5AwzfYdygsy8MD-kj530BYx9up9AJEZN_cnvI

11 We thank an anonymous reviewer for this insight.

12 Instead of treating the variables of trust differentials as continuous, we recode them into categorical ones. Specifically, we created a three-category variable to indicate negative values, 0, and positive values for each variable of trust differential. Using the negative values as the baseline category, we estimate models analogous to those in Table 1. The key results of this operationalization, as reported in Table A.5 and Figure A.1 in the supplementary material, remain similar to those in Table 1 and Fig. 2.

13 We also replace the US with Germany in our estimation. Our key results, as reported in Sect. 6 in the supplementary material, remain unchanged.
