# Supplementary material for: WHO approves? Relative trust, the WHO, and China’s COVID-19 vaccines
Source: Rev Int Organ. 2022 Nov 21:1–23. Online ahead of print. doi: 10.1007/s11558-022-09481-1 (PMC9684827; doi:10.1007/s11558-022-09481-1)
Supplement: Supplementary file 1 — Supplementary file1 (DOCX 267 KB) [file 11558_2022_9481_MOESM1_ESM.docx]

**Supplementary Material for**

**WHO Approves? The WHO’s Credibility and China’s COVID-19 Vaccines**

**Contents**

[1. Operationalization of Variables 2](#_Toc116600358)

[2. Summary Statistics 3](#_Toc116600359)

[3. Randomization Check 4](#_Toc116600360)

[4. Support for Government Procurement of COVID-19 Vaccines 5](#_Toc116600361)

[5. Transcription of Survey Questionnaire 6](#_Toc116600362)

[(1) Survey Procedure 6](#_Toc116600363)

[(2) Survey Structure 6](#_Toc116600364)

[(3) Survey Questionnaire 7](#_Toc116600365)

[6. Additional Analysis 9](#_Toc116600366)

# Operationalization of Variables

Table A.1 describes the operationalization of variables used in the empirical analysis of this study.

**Table A.1. Operationalization of Variables**

| **Variables** | **Coding** |
| --- | --- |
| Support for Government Procumbent of COVID-19 Vaccines from Abroad | Respondents’ support for government procurement of COVID-19 vaccines from abroad. 0: strongly do not support or somewhat do not support; 1: somewhat support or strongly support 0. |
| Age | Respondents’ age in years |
| Female | Respondents’ gender: 0: male; 1: female. |
| College | Coded as 1 if respondents have a college degree; otherwise as 0. |
| Willingness to Take Vaccines | (On a 0 to 10 scale) Respondent’s willingness to take COVID-19 vaccines: 0: definitely will not take; 10: definitely will take. |
| Support for Incumbent | Respondents’ party identification. 0: identified with opposition parties or non-partisanship; 1: identified with the incumbent |
| Nationalism | Respondents’ level of agreement with the following statement: “The world would be a better place if people of other countries behave like people of our country.” 1: Strong disagree; 2: Somewhat disagree; 3: Somewhat agree; 4: Strongly agree. |
| WHO Approval | Dummy variable for experiment groups: 0: No mention of WHO Approval; 1: Indication of WHO approval |
| Trust in WHO/China/US/Germany | Trustworthiness of the WHO/China/the US/Germany: 1: very untrustworthy; 2: somewhat untrustworthy; 3: Somewhat trustworthy; 4: very trustworthy. |
| Transparency-WHO/China/US/Germany | Transparency in releasing information about COVID-19: 1: Not transparent at all; 2: Somewhat not transparent; 3: Somewhat transparent; 4: Very transparent. |
| Performance-WHO/China/US/Germany | Performance of addressing the COVID-19 pandemic: 1: Very bad; 2: Bad; 3: Good; 4: Very Good |

# Summary Statistics

Table A.2 describes the summary statistics of variables used in this study.

**Table A.2. Summary Statistics**

| **Variable** | **N** | **Mean** | **Std. dev.** | **Min.** | **Max.** |
| --- | --- | --- | --- | --- | --- |
| Support for Gov. Procurement (%) | 950 | 67 | 47 | 0 | 100 |
| Age | 950 | 38.81 | 11.44 | 20 | 65 |
| Female (%) | 950 | 49 | 50 | 0 | 1 |
| College (%) | 950 | 63 | 48 | 0 | 1 |
| Willingness to Take Vaccines | 950 | 6.68 | 2.76 | 0 | 10 |
| Support for Incumbent (%) | 950 | 14 | 35 | 0 | 1 |
| Nationalism | 950 | 2.67 | 0.81 | 1 | 4 |
| Trust in WHO | 904 | 2.15 | 0.82 | 1 | 4 |
| Trust in China | 899 | 1.66 | 0.76 | 1 | 4 |
| Trust in the US | 894 | 2.96 | 0.70 | 1 | 4 |
| Trust Differential (WHO – China) | 883 | 0.48 | 0.79 | -3 | 3 |
| Trust Differential (WHO – the US) | 875 | -0.82 | 1.03 | -3 | 3 |
| Trust Differential (WHO – Germany) | 820 | -0.92 | 0.98 | -3 | 3 |
| Transparency-WHO | 860 | 2.32 | 0.86 | 1 | 4 |
| Transparency-China | 907 | 1.67 | 0.83 | 1 | 4 |
| Transparency-US | 896 | 3.09 | 0.67 | 1 | 4 |
| Transparency-Germany | 778 | 3.13 | 0.69 | 1 | 4 |
| Transparency Differential (WHO – China) | 852 | 0.65 | 0.87 | -3 | 3 |
| Transparency Differential (WHO – the US) | 846 | -0.77 | 0.96 | -3 | 3 |
| Transparency Differential (WHO – Germany) | 758 | -0.80 | 0.97 | -3 | 3 |
| Performance-WHO | 880 | 2.05 | 0.82 | 1 | 4 |
| Performance-China | 890 | 2.01 | 0.96 | 1 | 4 |
| Performance-US | 887 | 2.53 | 0.77 | 1 | 4 |
| Performance-Germany | 789 | 2.82 | 0.70 | 1 | 4 |
| Performance Differential (WHO – China) | 857 | 0.04 | 0.92 | -3 | 3 |
| Performance Differential (WHO – the US) | 858 | -0.48 | 0.90 | -3 | 3 |
| Performance Differential (WHO – Germany) | 776 | -0.76 | 0.89 | -3 | 2 |

# Randomization Check

Table A.3 reports the balance table of variables in this study.

**Table A.3. Balance Table**

| **Variable** | **All** | **China** | **China &** | **Foreign** | **Foreign &** |
| --- | --- | --- | --- | --- | --- |
|  |  |  | **WHO** |  | **WHO** |
| Support for Gov. Procurement (%) | 67 | 38 | 48 | 96 | 85 |
| Age | 38.81 | 38.16 | 39.74 | 38.28 | 39.08 |
| Female (%) | 49 | 49 | 45 | 57 | 45 |
| College (%) | 63 | 61 | 60 | 67 | 65 |
| Willingness to Take Vaccines | 6.68 | 7.09 | 6.46 | 6.61 | 6.55 |
| Nationalism | 2.68 | 2.68 | 2.68 | 2.73 | 2.62 |
| Support for Incumbent (%) | 14 | 13 | 13 | 14 | 15 |
| Trust in WHO | 2.15 | 2.15 | 2.20 | 2.12 | 2.15 |
| Trust in China | 1.66 | 1.75 | 1.61 | 1.69 | 1.59 |
| Trust in the US | 2.96 | 3.00 | 2.97 | 2.93 | 2.95 |
| Trust in Germany | 3.13 | 3.10 | 3.06 | 2.98 | 3.09 |
| Transparency-WHO | 2.31 | 2.39 | 2.32 | 2.32 | 2.24 |
| Transparency-China | 1.67 | 1.76 | 1.58 | 1.67 | 1.66 |
| Transparency-US | 3.08 | 3.12 | 3.12 | 3.03 | 3.09 |
| Transparency-Germany | 3.14 | 3.14 | 3.18 | 3.07 | 3.15 |
| Performance-WHO | 2.05 | 2.04 | 2.10 | 2.05 | 2.02 |
| Performance-China | 2.01 | 2.04 | 1.99 | 2.02 | 2.00 |
| Performance-US | 2.53 | 2.53 | 2.53 | 2.47 | 2.58 |
| Performance-Germany | 2.82 | 2.81 | 2.83 | 2.78 | 2.84 |

Note: Numbers in cells are mean values.

# Support for Government Procurement of COVID-19 Vaccines

Table A.4 reports the distribution of respondents’ support for government procurement of different COVID-19 vaccines by their developers and WHO approval.

**Table A.4. Support for Government Procurement of COVID-19 Vaccines**

| **Experimental Groups** | **Respondents Answers** | | | |  |
| --- | --- | --- | --- | --- | --- |
|  | Strongly Do | Somewhat Do | Somewhat | Strongly | **Total** |
|  | Not Support | Not Support | Support | Support |  |
| Chinese Vaccines | 58 | 89 | 70 | 21 | 238 |
|  | (24.37) | (37.39) | (29.41) | (8.82) | (100) |
| Chinese Vaccines + | 46 | 78 | 91 | 23 | 238 |
| WHO Approval | (19.33) | (32.77) | (38.24) | (9.66) | (100) |
| Foreign Vaccines | 1 | 9 | 114 | 112 | 236 |
|  | (0.42) | (3.81) | (48.31) | (47.46) | (100) |
| Foreign Vaccines + | 11 | 24 | 130 | 73 | 238 |
| WHO Approval | (4.62) | (10.08) | (54.62) | (30.67) | (100) |
| Total | 116 | 200 | 405 | 229 | 950 |
|  | (12.21) | (21.05) | (42.63) | (24.11) | (100) |

Note: Numbers in parentheses are row percentages.

# Transcription of Survey Questionnaire

# Survey Procedure

We conducted a survey experiment involving a diverse sample of 950 Taiwanese adults from May 23 to June 6, 2021. The sample was recruited by Rakuten Insight, an international public opinion company that conducts online surveys in Asian countries. Our sample was recruited to reflect the composition of the adult population of Taiwan in terms of gender, age, and geographic distribution.

# Survey Structure

Our survey was divided into three parts. First, the subjects were asked demographic questions, including their gender, year of birth, education level, and residence area. Second, they were asked questions designed to investigate their attitudes toward COVID-19 and their overall level of trust in the WHO and five countries, namely, Taiwan, the US, Germany, Russia, and China. We also asked them to evaluate the overall performance of the WHO and these countries in containing the COVID-19 pandemic.

The third part of the survey consisted of the experiment. We began by asking respondents about their willingness to be vaccinated against COVID-19 on a 0-10 scale, with a higher number indicative of more willingness. We then randomly assigned each respondent to one of the four experimental groups under different vignettes, which varied in terms of the origins of the COVID-19 vaccines and whether the WHO had approved them. Group 1, the “foreign vaccines group,” were asked to read the following paragraph. The questionnaire is displayed in the following pages:

# Survey Questionnaire

**Part I: Questions on Democratic Characteristics**

**Q1: What is your gender?**

1. Male; (2) Female

**Q2: What is your highest level of education?**

1. No formal education; (2) Elementary school; (3) Junior high school; (4) Senior high school; (5) College or junior college; (6) Graduate school or above.

**Q3: What is your birth year?**

**Q4: What is your place of residence?**

**Q5: What party do you support?**

**Party II: Basic Questions on COVID-19**

**Q6: If 0 means that you would not consider taking Covid-19 vaccines and 10 means that you definitely take Covid-19 vaccines, where would you put yourself on this scale?**

**Q7: Do you agree with the following statement?  “The world would be a better place if people of other countries behave like people of our country.”**

1. Strong disagree; (2) Somewhat disagree; (3) Somewhat agree; (4) Strongly agree.

**Q8: What is your opinion on the following different governments’ and international organizations’ transparency in releasing information about the Covid-19 pandemic (Taiwan; The US; Germany; China; Russia; and the WHO?)**

1. Not transparent at all; (2) Somewhat not transparent; (3) Somewhat transparent; (4) Very transparent

**Q9: What is your opinion on the different governments’ and international organizations’ performance in addressing the Covid-19 outbreak (Taiwan; The US; Germany; China; Russia; and the WHO?)**

1. Very bad; (2) Bad; (3) Good; (4) Very good.

**Q10: What are your levels of trust in the following countries and international organization (Taiwan; The US; Germany; China; Russia; and the WHO?)**

1. Not trustworthy at all; (2) Somewhat not trustworthy; (3) Somewhat trustworthy; (4) Very trustworthy.

**Party III: Experimental Vignette**

**Q11a: Scientists have developed COVID-19 vaccines, and our government is beginning to procure COVID-19 vaccines from abroad for your fellow countrymen. Do you support procurement by our government of COVID-19 vaccines developed by foreign countries?**

1. Strongly do not support; (2) Somewhat do not support; (3) Somewhat support; (4) Strongly support.

**Q11b: Scientists have developed COVID-19 vaccines, and our government is beginning to procure COVID-19 vaccines from abroad for your fellow countrymen. Do you support procurement by our government of COVID-19 vaccines developed by China?**

1. Strongly do not support; (2) Somewhat do not support; (3) Somewhat support; (4) Strongly support.

**Q11c: The World Health Organization (WHO) is using COVID-19 Vaccines Global Access (COVAX) to provide countries with COVID-19 vaccines that it has approved. Do you support procurement by our government of COVID-19 vaccines developed by foreign countries and approved by the WHO?**

1. Strongly do not support; (2) Somewhat do not support; (3) Somewhat support; (4) Strongly support.

**Q11d: The World Health Organization (WHO) is using COVID-19 Vaccines Global Access (COVAX) to provide countries with COVID-19 vaccines that it has approved. In May 2021, the WHO approved the Sinopharm vaccine developed in China and included it in COVAX. Do you support procurement by our government of China’s COVID-19 vaccines approved by the WHO?**

1. Strongly do not support; (2) Somewhat do not support; (3) Somewhat support; (4) Strongly support.

# Additional Analysis

Instead of treating the variables of trust differentials as continuous, we recode them into categorical ones. Specifically, we created a three-category variable to indicate negative values, 0, and positive values for each variable of trust differential. Using the negative values as the baseline category, we estimate four models analogous to those in Table 1. The key results of this operationalization, as reported in Table A.5 and Figure A.1 below, remain similar to those in Table 1 and Figure 2 in the main text.

**Table A.5: Estimates of Support for Government Procurement of COVID-19 Vaccines from Abroad**

|  | **Model 1** | **Model 2** |  | **Model 3** | **Model 4** |
| --- | --- | --- | --- | --- | --- |
|  | Chinese Vaccines | |  | Foreign Vaccines | |
| Age | 0.020* | 0.021* |  | 0.000 | 0.001 |
|  | [0.010] | [0.010] |  | [0.014] | [0.014] |
| Female | -0.318 | -0.321 |  | 0.406 | 0.404 |
|  | [0.217] | [0.217] |  | [0.365] | [0.366] |
| College | 0.086 | 0.078 |  | 0.771* | 0.787* |
|  | [0.223] | [0.224] |  | [0.371] | [0.388] |
| Willingness | -0.013 | -0.013 |  | 0.227*** | 0.228*** |
|  | [0.042] | [0.042] |  | [0.067] | [0.068] |
| Support Incumbent | -1.321*** | -1.290*** |  | -0.592 | -0.597 |
|  | [0.372] | [0.374] |  | [0.485] | [0.489] |
| Nationalism | -0.565*** | -0.567*** |  | -0.012 | -0.008 |
|  | [0.128] | [0.129] |  | [0.266] | [0.265] |
| WHO Approval | 0.341 | -0.699 |  | -1.444*** | -1.650** |
|  | [0.208] | [0.886] |  | [0.406] | [0.534] |
| Trust Differentials (WHO－China = 0) | -0.366 | -0.652 |  |  |  |
|  | [0.458] | [0.572] |  |  |  |
| Trust Differentials (WHO－China > 1) | -0.525 | -1.060+ |  |  |  |
|  | [0.463] | [0.588] |  |  |  |
| Trust Differentials (WHO – China = 0) |  | 0.873 |  |  |  |
| X WHO Approval |  | [0.929] |  |  |  |
| Trust Differentials (WHO – China > 1) |  | 1.348 |  |  |  |
| X WHO Approval |  | [0.941] |  |  |  |
| Trust Differentials (WHO－US = 0) |  |  |  | 0.609 | 0.228 |
|  |  |  |  | [0.449] | [0.841] |
| Trust Differentials (WHO－US > 1) |  |  |  | 0.061 | -0.641 |
|  |  |  |  | [0.593] | [0.975] |

*(Continued on the next page)*

**Table A.5: Continued**

|  | **Model 1** | **Model 2** |  | **Model 3** | **Model 4** |
| --- | --- | --- | --- | --- | --- |
|  | Chinese Vaccines | |  | Foreign Vaccines | |
| Trust Differentials (WHO－US = 0) |  |  |  |  | 0.500 |
| X WHO Approval |  |  |  |  | [1.033] |
| Trust Differentials (WHO－US > 1) |  |  |  |  | 0.899 |
| X WHO Approval |  |  |  |  | [1.192] |
| Constant | 0.964 | 1.290+ |  | 1.197 | 1.307 |
|  | [0.707] | [0.773] |  | [1.226] | [1.238] |
| Log pseudolikelihood | -272 | -271 |  | -113 | -113 |
| No. of Observations | 441 | 441 |  | 438 | 438 |

Note: Robust standard error in brackets. + 0.1, * p < 0.05, ** p < 0.01, *** p < 0.001. All tests are two-tailed.

Based on the estimation results of Model 2 and Model 4 in Table A.5, Figure A.1 illustrates the marginal effects of WHO approval on support for government procurement of vaccine from abroad conditional on respondents’ trust differentials between the WHO and China and between the WHO and the US. The patterns in both panels of Figure A.1 are similar to Figure 2 in the main text.

**Figure A.1: Marginal Effect of WHO Approval on Support for Government Procurement of Vaccine from Abroad across Trust Differentials**


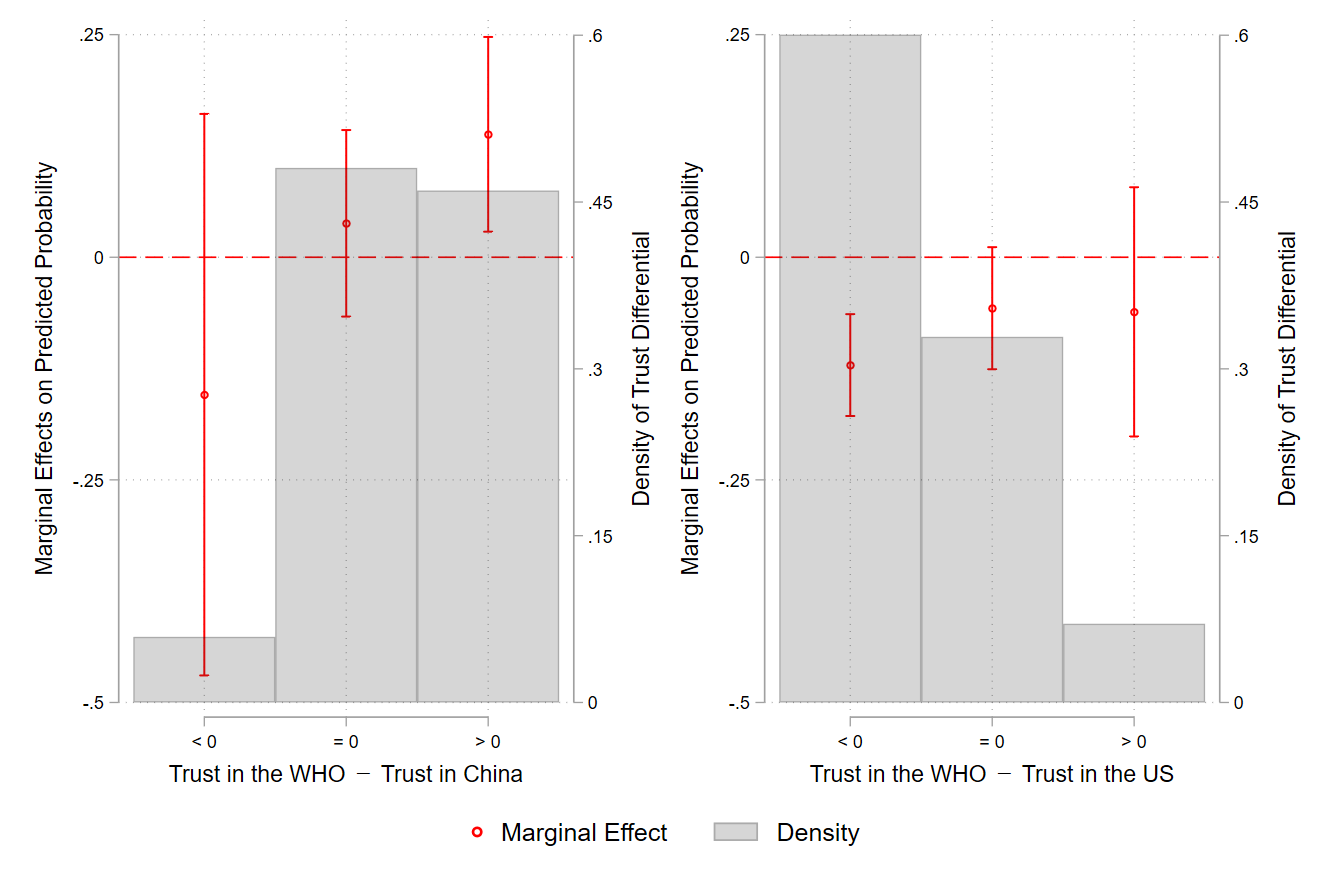


Table A.6 compares the results of respondents’ trust in the US to that of their trust in Germany as a proxy of their trust in foreign countries. The differences are statistically insignificant either between Model 1.1 and Model 2.1 or between Model 1.2 and Model 2.2.

**Table A.6: Estimates of Support for Government Procurement of COVID-19 Vaccines from Abroad**

|  | **Model 1.1** | **Model 1.2** |  | **Model 2.1** | **Model 2.2** |
| --- | --- | --- | --- | --- | --- |
|  | **US** | |  | **Germany** | |
| Age | -0.000 | 0.000 |  | -0.001 | -0.003 |
|  | [0.014] | [0.014] |  | [0.015] | [0.015] |
| Female | 0.398 | 0.407 |  | 0.594 | 0.568 |
|  | [0.376] | [0.370] |  | [0.393] | [0.383] |
| College | 0.744* | 0.775* |  | 0.760* | 0.773* |
|  | [0.367] | [0.374] |  | [0.371] | [0.374] |
| Willingness | 0.228*** | 0.235*** |  | 0.261*** | 0.259*** |
|  | [0.067] | [0.069] |  | [0.065] | [0.064] |
| Support Incumbent | -0.598 | -0.629 |  | -0.713 | -0.755 |
|  | [0.490] | [0.494] |  | [0.502] | [0.509] |
| Nationalism | 0.005 | 0.010 |  | -0.104 | -0.099 |
|  | [0.272] | [0.270] |  | [0.257] | [0.252] |
| WHO Approval | -1.455*** | -1.052* |  | -1.617*** | -1.203* |
|  | [0.410] | [0.501] |  | [0.408] | [0.498] |
| Trust Differential | 0.219 | -0.131 |  |  |  |
| (WHO－the US) | [0.181] | [0.326] |  |  |  |
| WHO Approval X Trust |  | 0.451 |  |  |  |
| Differential (WHO－US) |  | [0.400] |  |  |  |
| Trust Differential |  |  |  | 0.159 | -0.270 |
| (WHO－Germany) |  |  |  | [0.195] | [0.458] |
| WHO Approval X Trust |  |  |  |  | 0.517 |
| Differential (WHO－Germany) |  |  |  |  | [0.508] |
| Constant | 1.567 | 1.158 |  | 1.703 | 1.458 |
|  | [1.211] | [1.255] |  | [1.223] | [1.184] |
| Log pseudolikelihood | -114 | -113 |  | -106 | -105 |
| No. of Observations | 438 | 438 |  | 409 | 409 |

Note: Robust standard error in brackets. + 0.1, * p < 0.05, ** p < 0.01, *** p < 0.001. All tests are two-tailed.

Based on the estimation results of Model 1.2 and Model 2.2 in Table A.5, Figure A.2 illustrates the marginal effects of WHO approval on support for government procurement of vaccine from abroad conditional on respondents’ trust differentials between the WHO and the US and between the WHO and Germany. Both panels of Figure A.1 suggest no significant difference between using respondents’ trust in the US and their trust in Germany as the proxy of their trust in foreign countries.

**Figure A.2: Marginal Effect of WHO Approval on Support for Government Procurement of Vaccine from Abroad across Trust Differentials**

| 1. Using Trust in the US as a Proxy | 1. Using Trust in Germany as a Proxy |
| --- | --- |
| 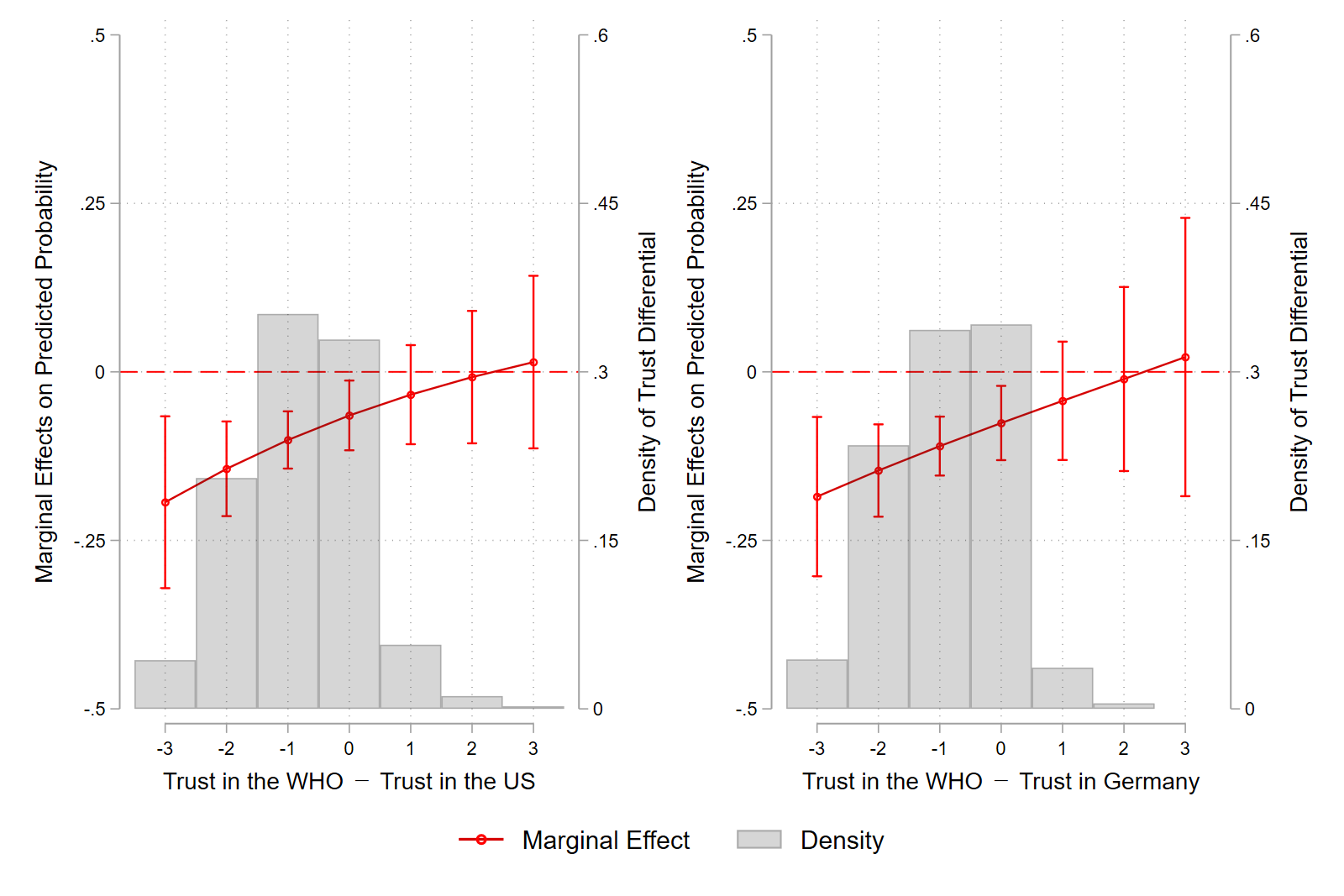 | |
